# On the Design of a Bioacoustic Sensor for the Early Detection of the Red Palm Weevil

**DOI:** 10.3390/s130201706

**Published:** 2013-01-30

**Authors:** Miguel Martínez Rach, Héctor Migallón Gomis, Otoniel López Granado, Manuel Perez Malumbres, Antonio Martí Campoy, Juan José Serrano Martín

**Affiliations:** 1 Department of Physics and Computer Science, Miguel Hernandez University, Ave. Universidad s/n-Ed. Alcudia, 03202 Elche, Spain; E-Mails: mmrach@umh.es (M.M.R.); hmigallon@umh.es (H.M.G.); otoniel@umh.es (O.L.G.); 2 Department of Computer Engineering, Technical University of Valencia, Camino de Vera s/n, 46021 Valencia, Spain; E-Mails: amarti@disca.upv.es (A.M.C.); jserrano@itaca.upv.es (J.J.S.M.)

**Keywords:** pest detection, acoustic signal processing, wavelet transform, pattern matching, acoustic sensor design, wireless sensor networks

## Abstract

During the last two decades Red Palm Weevil (RPW, *Rynchophorus Ferrugineus*) has become one of the most dangerous threats to palm trees in many parts of the World. Its early detection is difficult, since palm trees do not show visual evidence of infection until it is too late for them to recover. For this reason the development of efficient early detection mechanisms is a critical element of RPW pest management systems. One of the early detection mechanisms proposed in the literature is based on acoustic monitoring, as the activity of RPW larvae inside the palm trunk is audible for human operators under acceptable environmental noise levels (rural areas, night periods, *etc.*). In this work we propose the design of an autonomous bioacoustic sensor that can be installed in every palm tree under study and is able to analyze the captured audio signal during large periods of time. The results of the audio analysis would be reported wirelessly to a control station, to be subsequently processed and conveniently stored. That control station is to be accessible via the Internet. It is programmed to send warning messages when predefined alarm thresholds are reached, thereby allowing supervisors to check on-line the status and evolution of the palm tree orchards. We have developed a bioacoustic sensor prototype and performed an extensive set of experiments to measure its detection capability, achieving average detection rates over 90%.

## 1. Introduction

Red palm weevil (RPW, *Rhynchophorus Ferrugineus* Oliv., (Curculionidae, Coleoptera)) is a serious pest that attacks different species of palm trees (e.g., date palm, coconut palm, and royal palm). The RPW pest was reported in Asia, Australia, Philippines, and Thailand as early as 1962 [1]. Since then, its expansion has covered near all countries in Asia, Middle East [2] and the Mediterranean Rim. Recently, the RPW pest has also been reported in different areas of the American continent, being currently considered as a global pest. This high rate of spread is largely caused by human intervention, by transporting infested young or adult date palm trees and offshoots from contaminated to uninfected areas. Date palm is an important crop in North African and Asian countries and ornamental palms are widely planted as amenity trees in the whole Mediterranean area.

This pest is especially destructive because visible symptoms only appear when the infestation is severe. By then, it is too late to save the palm tree, therefore, only preventive actions are really effective. Among these actions, early detection systems are crucial to fight against RPW pest, since they can quickly detect it in the early infestation stages and trigger the actuation protocol to save the rest of the plantation. After RPW detection, a deep inspection around the detection area is carried out, to destroy the severely infested trees, evaluate those endangered trees to determine its treatment and biological traps deployed. This protocol prevents the rest of the plantation from being infested, so an effective early detection system is fundamental to save as many palm trees as possible, working as a defensive protection barrier. In [3] the authors expose an extensive compilation of works related to the RPW pest, examining in detail several aspects of the problem as its historical evolution, RPW biological cycle, economic aspects derived from RPW pest, pest management strategies, *etc.*

Different technologies have been applied to detect the initial stages of RPW pest infestation. In [4] the authors employ a Computer Assisted Tomography system for the inspection of infested wheat, obtaining good results. However, their proposal has two main drawbacks that limit its applicability: (1) use of a very expensive system, and (2) the difficulty of scanning adult palm trees *in-situ*, since the X-Ray device requires special power supplies. Other trials were based in the use of gas sensors, in order to detect the characteristic smell of some volatiles, generated during the fermentation processes in the infested palms, as some previous experiences using trained dogs had good results [5]. Anyway, gas sensors are not very selective and their response is influenced by many other volatiles, as shown at [6].

Most of the works about RPW early detection systems are related to acoustic sensors, because the activity of RPW larvae inside the palm trunk is audible for human operators under acceptable environmental noise levels (rural areas, night periods, *etc.*). In [7] and [8], some acoustic sensors specifically designed to detect RPW activity have been proposed. They employ an acoustic probe inserted in the palm trunk to improve the capture of sounds made by the RPW larvae. Then, the sensor device analyzes the captured sound in real-time, supplying an audible tone when the analysis detects a sound. The sensor is managed by a trained operator who, depending on the number and frequency of the positive tones, determines whether the palm tree is infested. In [9], another RPW bioacoustic sensor is provided to analyze the audio captured from the interior of a palm trunk, which is the same idea than the one proposed in [7]. But, instead of audible tones, their device activates a blinking red LED to indicate the presence of RPW activity. In this case, the operator training is not required. Among the experimental results, authors have proved that, under controlled environmental conditions, it is possible to detect acoustically two week old larvae activity in palms infested with only five individuals.

A portable acoustic device is proposed in [10] for the RPW on-line detection. It processes the audio signal captured from the palm, applying an active band pass filter in the 800–2,500 Hz frequency band, which has been identified as the effective frequency range of the red palm weevil's acoustic emissions. The device produces a clip sound when RPW activity is found, then the human operator can decide whether the monitored palm is infested. The accuracy reported by authors is around 97% with measurements collected at four different points in each palm.

In [11] the authors propose a signal processing system to detect the presence of RPW by defining an extensive set of temporal features (signal roll-off, slope and temporal spread) and tuning processing parameters as optimum frame size and proper window functions. Additional related works, like [12] and [13], perform detailed studies about RPW sound activity when moving and feeding, and identifies some spectral and temporal features of RPW sound activity. In particular, they analyze the sound impulse bursts from RPW feeding activities in order to isolate them from other audio sources (wind, birds, *etc.*).

In [14], the authors propose a unified acoustic detection system for specific insect pests, analyzing both the RPW and Rice Wheevil (RW) pests as two particular cases. The proposed signal detection system employs matching learning methods, based on a particular set of signal features to properly classify the captured audio signals. This approach is able to achieve 99.1% and 100% detection performance rates with RPW and RW, respectively. These results have been obtained with real-field recordings over a laptop computer, but no computational complexity of the detection system is reported and a reduced human *in-situ* presence is still required.

A remarkable acoustic detection system is proposed in [15], where authors use a mathematical method for automatic detection of RPW acoustic activity in offshoots. The methodology applied is similar to some techniques used in the field of speech recognition, utilizing Vector quantization (VQ) or Gaussian mixture modeling (GMM). The proposed algorithm successfully achieves detection ratios as high as 98.9%. These are very good results but, as authors state in their work, they have been obtained under optimal conditions: the recordings were taken inside a sound-proof recording box in a noiseless environment.

All the approaches mentioned above require in-situ monitoring, thus, for extensive monitoring of large plantations, these proposals are time, labor and cost-consuming activities. In addition, the monitoring process is developed at one particular period of time, that is to say, there is no continuous monitoring of palm trees. This fact would make the detection delay highly dependent on the monitoring frequency (the number of monitoring processes performed in a period of time), taking also into account that the higher the monitoring frequency is, the higher the overall monitoring costs.

Consequently, we are interested in specific bioacoustic sensors that may be physically installed in the supervised palm trees to perform continuous monitoring. This approach reduces significantly the overall monitoring costs and the detection delay. The desired bioacoustic sensors are designed to be autonomous devices (battery operated) with large operational working lives (greater than one year) and a wide range of monitoring frequencies.

In this paper, we design and develop a bioacoustic sensor prototype which efficiently detects the sounds produced by RPW larvae after the first infestation stages. The proposed bioacoustic sensor is able to: (1) effectively detect the RPW presence with high detection rates (over 90%); (2) perform monitoring tests at user programmable frequencies in order to achieve a fast detection response; (3) work autonomously during large periods of time (at least one year); (4) work without maintenance requirements after installation; (5) form a wireless sensor network to cover from little orchards to large plantation extensions; (6) allow continuous monitoring activity, since data may be checked on-line in real-time through an Internet connection with the control station; (7) trigger an alarm system defined at the control station to warn supervisors about the desired events by means of e-mail, Short Message System (SMS), Instantaneous Message System (IMS), *etc.*

This device is composed by an audio probe inserted into the palm trunk to record the sounds produced inside. The audio probe is connected to the sensor board, where the captured sounds are processed in real-time, to determine the presence of RPW larvae activity. The sensor board is equipped with a wireless communication interface which periodically sends the monitoring results to a control station. At control station, the reports received from installed sensors are further processed and conveniently stored with the corresponding side information (palm ID, geolocation info, timestamp, report summary, *etc.*). If the control station has available Internet access, all received data can be accessed remotely, and some alarm settings can be configured to proactively send alert messages to supervisors (RPW presence, node failure, *etc.*). In this paper we only cover the design and evaluation of the bioacoustic sensor prototype; therefore, networking issues, control station functionality, and extensive field experiments are part of our future work.

## 2. Bioacoustic Sensor: Hardware Description

**Figure 1.**
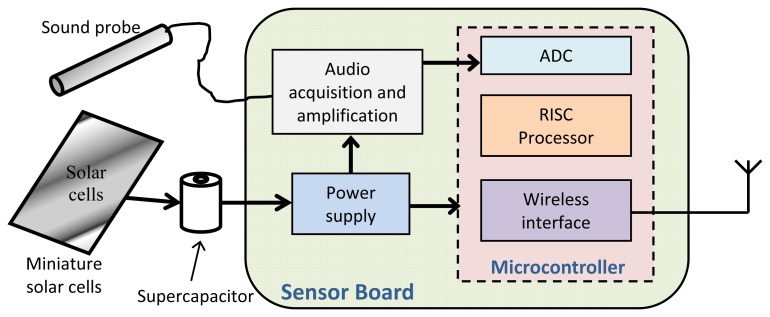
Block diagram of the proposed RPW bioacoustic sensor.

In this section we provide details about the design of our bioacoustic sensor. The main components of the proposed sensor architecture, shown at Figure 1, are the following:

- An audio probe, in charge of acquisition of sounds from the RPW, conditioning and properly amplifying the captured audio signal, making it suitable to be processed by the detection algorithm.
- A low-power processor, that will be able to run the detection algorithm to process the sound captured by the audio probe and determine the RPW presence.
- A wireless communication interface, able to deliver data messages reporting the results of RPW activity.
- A power supply unit, in charge to provide suitable power to the bioacoustic sensor node.

The audio probe is a specific device designed to work in the analog domain and to provide the signal captured from the palm tree with the highest possible quality. The design and the details of the audio probe are presented in the following section.

Regarding the processor element, it has to achieve some requirements: (1) it has to be able to digitalize, or get already digitalized, the audio signal provided by audio probe; (2) it has to be able to execute, in real-time, the proposed detection algorithm to detect the presence of RPW activity; (3) it has to be able to send messages using the available wireless network interface; (4) the processor element should present saving-energy or low-power characteristics, in order to work with batteries for a long time; (5) the expected weight and size of the bioacoustic sensor must be small enough to be installed at the top of a palm tree.

The last two requirements preclude the use of personal computers, PC-based industrial computers, and laboratory instrumentation. Low power DSP and microcontrollers meet all these requirements; thus, the first step has been to decide which device we should employ to build the core of the bioacoustic sensor. The chosen device has been a Jennic (now NXP) brand microcontroller, model JN5148-001 [16]. The wireless JN5148-001 microcontroller fits the requirements of computation power, networking, battery operation, size and audio acquisition demanded by the RPW application. Its main characteristics are:

- Low power operation modes, from 1.5 μA in sleep mode, and close to 200 mA in full power mode with all peripherals working on.
- Thirty two-bits RISC pipelined processor.
- One memory of 128 KB ROM and one memory of 128 KB RAM, large enough to store program and data used to perform the bioacoustic detection of the RPW.
- Wireless 2.4 GHz, IEEE 802.15.4 compliant transceiver, with ZigBee network support.
- Up to 21 general purpose digital inputs/outputs.
- Twelve bit ADC with a maximum sampling frequency of 100 kHz.
- Four wire interface for digital audio.

With reference to the audio acquisition task, there are two possible ways: First, using the four wire audio interface; and second, using the Analog-to-Digital Converter (ADC). With the first option, the use of an external audio Coder/Decoder (CODEC), including an ADC and communications hardware, is mandatory. Some CODECs include an amplification stage, which may be an advantage. With the second option, the audio conditioning and implementation should be done by means of a self-designed analog circuit, using the on-chip ADC of the microcontroller to digitalize the audio. The advantage of this option is that parameters and power consumption of the amplification stage may be fine-grain designed, so we have decided to use this approach in our bioacoustic sensor.

Regarding the power supply, the bioacoustic sensor measured consumption rises up to 200 mA when performing detection process. Although detection may be decomposed in four stages–sound acquisition, digitalization, audio analysis and transmission of results- the detection algorithm works in real-time, hence, all devices for signal conditioning, analog-to-digital conversion, and execution of the detection algorithm, must run simultaneously. Radio communication proceeds at the end of the detection process, therefore, at this point consumption keeps below maximum because the analog stage, including analog-to-digital converter, is switched off.

Despite this high power requirement, the bioacoustic sensor is not working full-time. As explained below, the sensor node works in a predefined duty cycle, where most of the time it stays in an ultra-low power consumption mode (sleep mode). When sensor wakes up from sleep mode, it enters running mode, where all the required systems are turned on, and the RPW detection software starts to work during a limited period of time (between 5 and 10 minutes, although it may be programmable). At the end of running mode, the sensor node wirelessly sends the corresponding RPW activity report found during the capture session. This working behaviour is periodically repeated during the life of the sensor node, performing between 3 and 12 audio analysis a day (again this may be modified). In the best case, a battery of 2,000 mAh works for no more than one month. The use of batteries with more capacity is precluded because of its high weight and size and the common location of the sensor, on top of palm trees.

To increase the operating time of the bioacoustic sensor, we propose a power unit based on the use of a supercapacitor, which usually has less charge than a battery, but is rechargeable. The supercapacitor is able to supply a maximum current load of 500 mA during one hour of continuous operation before running out of energy. In order to recharge the supercapacitor, we use a set of eight miniature solar cells, arranged in parallel to gain enough current, about 350 mA. Every cell works at 3.0 volts and is able to give 45 mA, with dimensions: 54 mm × 43 mm × 3.0 mm (L × W × D). Obviously, power generation levels are lower on cloudy days; light cloud cover can reduce the output by as much as a half, and on a very overcast day it drops to as little as 5%–10%. However, this level of energy generation is still enough to meet the supercapcitor charging requirements.

The current produced by solar cells is supplied to a Low Dropout (LDO) Linear Voltage Regulator (LT3085 from Linear Technology), to adjust the solar cells voltage to the input range demanded by supercapacitor, which operates at 2.3 volts. The output of the supercapacitor powers the sensor board using a Step-Up Voltage Regulator (L6920 from STMicroelectronics) to properly adjust capacitor's voltage to that required by the sensor board. Also, a conventional, non-rechargeable backup battery is included to supply power to the bioacoustic sensor when there is no solar light and the supercapacitor is not capable to power up the system.

**Figure 2.**
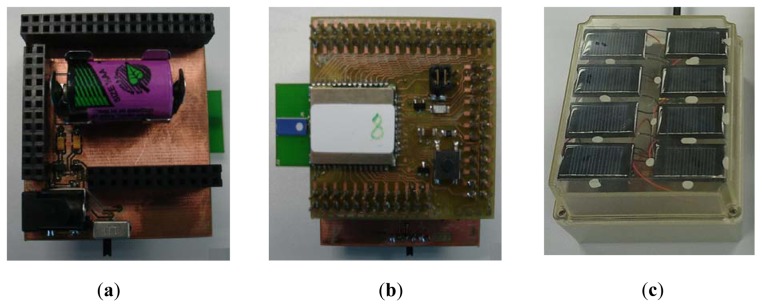
Bioacoustic sensor node prototype (**a**) upper side view, (**b**) down side view, and (**c**) solar-based power unit.

Finally, the radio interface is up to create a reliable point-to-point outdoor communication over distances of 30 meters. Nominal values from manufacturer are larger, but they can be achieved only in optimal conditions of no EMI interferences, higher transmission power (up to 20 dBm), and particular antenna arrangement and orientation. The 30 meters maximum reliable range has been proposed after performing some experimental field tests with two sensor prototypes in Line Of Sight (LOS), urban scenarios and low power transmission profiles. In Figure 2 some pictures about the RPW bioacoustic sensor prototype are shown. We can observe both sides of the sensor board prototype and the solar-based power supply we use to power our sensor.

## 3. Bioacoustic Sensor: Audio probe

The audio probe is composed by three elements: the microphone, the probe and the signal conditioning stage. To decide the appropriate audio probe design, we have analyzed the features of RPW sound: (a) studying previous works like [11–13], (b) performing a preliminary spectral and temporal analysis of available RPW audio recordings, and (c) taking into account several aspects, as probe insertion into palm trees, environmental noise levels, signal adjust conditioning parameters (*i.e.*, signal amplification and filtering) among others, by means of laboratory and field tests. Below, we explain the details that define each probe element, its multiple design approaches and the reasons that have led us to choose the proposed design.

### 3.1. Microphone

Regarding the microphone, we have considered two alternatives: MEMS or silicon microphones and electret microphones. The major advantage of MEMS microphone is its small size. With this kind of microphone the attachment to palm tree is very easy and of low injury, both using inserting nails/probes or surface sand down allocation. However, mass diaphragm of MEMS is small, so the sensitivity to vibrations is really low. Experiences were accomplished with Analog Devices ADMP401 and ADMP405, with sensitivities of −42 dB and −38 dB at 1 kHz, respectively, and 3.35 × 2.5 × 0.88 mm size.

**Figure 3.**
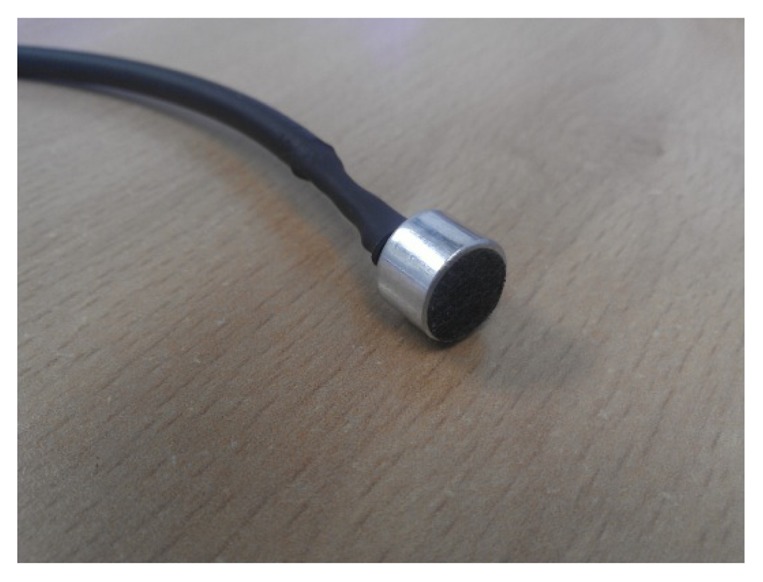
MCE-100 Microphone.

For electret microphones it is possible to find a large range of sizes and sensitivities. The behavior is similar to MEMS microphones—sensitivity, linearity, range of frequencies, but the main difference is the size. This kind of microphones, with cylinder shape, may range from 2 mm up to 10 mm of diameter and length between 2 mm and 7 mm. Experiences were accomplished with PRO-SIGNAL MCE-100 (see Figure 3) and MCE-400, both with an external diameter of 9.7 mm and length of 6.7 mm, and sensitivity of 5.6 mV/Pa and 7.9 mV/Pa at 1 kHz. We used also KINGSTATE KECG2740PBJ, a 6 mm external diameter and length of 2.7 mm, with a sensitivity of −40 dB at 1 kHz. All electret and MEMS microphones present similar degree of linearity at a frequency range between 100 Hz and 10 kHz, which it is enough for the RPW sound acquisition [7].

### 3.2. Acoustic Probe

To attach the sound sensor to the palm tree, we have considered two different probe designs. The first one consists of attaching the sensor to the palm tree surface. The main advantage of this approach is the low or even null impact in the palm tree structure. A 1 cm diameter circle has been sanded down to accommodate the sensor, which is held with a strap around the palm tree. Early experiments have shown that it is very hard to fix the sensor close to the stem. Also, this attaching method leaves the sensor exposed to environmental noise, which in urban gardens and parks may be higher than sounds coming from the palm tree trunk.

The second alternative was to use a nail to insert the sensing device inside the palm tree. This option provides two advantages: The sensor is closer to the sound source and it is isolated from external noise. The main disadvantage is the injury caused to the palm tree, leaving a 1 cm diameter hole; it requires being extremely careful when removing the probe, filling the affected area with appropriate putty material and/or painting it with a fungicide.

We have considered nails of plastic, copper and aluminum. The probes have been built as hollow cylinders of 6, 8, 10 and 12 mm of internal diameter, and around 10 cm of length. We have made some tests in laboratory with palm tree stem sections, in order to assess de behavior of different probe materials and audio sensors. In the experiments, the probes were inserted in a palm stem and the electret and MEMS microphones were placed some times in the outer extreme and other times in the inner extreme of the probe, to assess the proper position of the audio sensor inside the probe. Sounds were artificially produced using iron and wood tools scratching inside the stem. After performing several experiments, the following conclusions were drawn: (1) plastic probe presents the worse sound transmission, so it has been discarded; (2) there is no considerable difference between copper and aluminum probes regarding sound transmission; (3) the greater the probe diameter, the greater the system response, if microphone and probe diameters are the same; (4) if the microphone does not fit properly into the probe because its diameter is smaller, the sound transmission is considerably worse. This was more noticeable with MEMS microphones, with no circular shape and therefore, less contact surface with the probe. Also, its small size makes it difficult to fit and attach it to the inner face of the probe, to get gain from probe vibrations; (5) when the microphone is allocated in the outer extreme of the probe, the sound transmission is better, because the probe acts like a resonance chamber, amplifying the sound.

**Figure 4.**
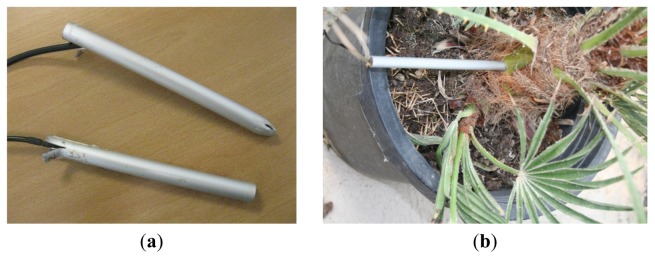
Open and Closed probes (**a**). Example of probe insertion (**b**).

Taking into account the obtained experimental results, we have decided to use an aluminum probe, because it is resistant to corrosion and easy to work, with a 10 mm of interior diameter, to get the maximum sound transmission and to avoid an excessive injury to the palm tree. Finally, the microphone has been situated in the outer side of the probe to get a better sound transmission, leaving the other end opened. After working with real palm trees, and in order to reuse the probes, we have decided to close the inserting end of the probe to prevent the entrance of palm grains and, at the same time, to protect the microphone. We have tested both approaches in laboratory, but no considerable differences were detected between audio signals captured with closed and open-ended probes. Figure 4(a) shows a picture of both open-end and closed-end audio probes, and, Figure 4(b) shows an example of probe insertion in a young palm.

Regarding the place of insertion of the probe, the RPW commonly starts infestation close to the top of the palm tree, so we suggest inserting the sound probe at the end of the stem which is located as close as possible to the palm crown. Some field experiences have showed us that it is more convenient to test first in the southern side of the palm tree side, because after surgery of infested palms, it may be appreciated that infestations begin in this side of the tree, but this fact should be formally studied.

### 3.3. Signal Conditioning: Filtering and Amplification Stage

Once we have defined the audio sensor and the probe, we need to perform some signal conditioning to deliver the microphone output signal to the A/D converter with the highest possible quality. The main issue here is the amplification stage, which is a well-known problem with lots of solutions. In this case, not all of these solutions are convenient, because the bioacoustic sensor has a set of requirements that limits the design: (1) it has to be powered by batteries, so the use of high and bipolar voltages should be avoided, and (2) sensor signal output has to be largely amplified, in the whole range of frequencies, up to 10 kHz.

To fit the requirements, we have used a Texas Instruments TLV2785 operational amplifier. This chip includes four operational amplifiers, with single supply since 1.8 V up to 3.6 V, and a supply current of less than 1 mA per channel, very convenient for battery operated devices. In addition, amplifiers may be shutdown to save battery when the device is not capturing sound. Its bandwidth is 8 MHz, allowing gains of more than 50 dB for a cut off frequency of 10 kHz.

**Figure 5.**
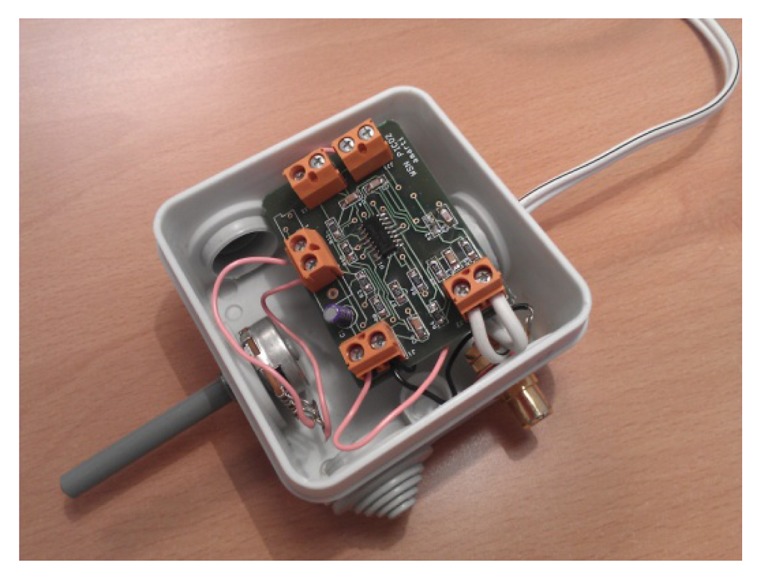
Signal conditioning and amplifying board prototype.

A PCB board has been designed, where two of the operational amplifiers are used to amplify the microphone output. Two additional amplifiers are used to create an active low pass filter at 10 kHz, to avoid aliases in the digital part of acquisition chain. This board is inserted in a plastic box, with an RCA connector for the microphone, a potentiometer to adjust the desired gain, and two jack outputs, one for the headphones and the other for the connection with an analog input of a microcontroller (a prototype is shown at Figure 5). The goal of this design is the modularity and flexibility of the device: different microphones may be connected to the amplifier, it can be used by a human operator listening through headphones, or it can be connected to the microcontroller for the autonomous detection.

### 3.4. Final Audio Probe Design

Taking into account all the different choices studied and tested, the acoustic probe final configuration is composed by: A 10 mm internal diameter aluminum probe with the inserting end closed, an MCE-100 electret microphone placed at the outer side of the probe and a high gain, high bandwidth operational amplifier.

## 4. Bioacoustic Sensor: Software Description

After having described the sensor board hardware architecture and the details about our specific RPW audio probe, we are going to explain the software installed in the sensor. The operating software is stored in the on-chip flash memory and is loaded at boot time. Just after a hardware reset or power on, the bioacoustic sensor enters the initialization stage. In practice, the initialization stage is done only once, when the sensor is installed for the first time.

During the bioacoustic sensor installation, the operator fixes the sensor (audio probe plus sensor board) to the palm tree at the desired monitoring location. Then, the sensor is configured by means of the Sensor Deployment Software (SDS) running in a portable device (smartphone, tablet, laptop, *etc.*) with GPS support and equipped with the control station (CS) radio interface (*i.e.*, a USB dongle) which allows direct communication with the sensor. This device acts as control station only during the sensor node initialization/configuration stage, without requiring the synchronization with other network nodes.

If the sensor node is not configured (first boot), it enters the configuration mode, waiting for the corresponding instructions. Then, the sensor deployment software delivers to the sensor node the configuration information, like node ID, GPS location, sensor node hardware address, sound capture configuration parameters (sample resolution, sampling rate, processing window size, *etc.*), audio analysis time interval, and the schedule for the first audio analysis. After receiving and storing the configuration data, the sensor node (1) configures the sensor board (wireless radio and peripherals), then (2) broadcasts the HELLO message indicating the end of the configuration mode, and finally (3) enters the standby mode to save power until the beginning of the first scheduled audio analysis.

After the installation of all the sensors, the information recorded by the SDS system is downloaded to the control station (ID, hardware address, GPS location, capture cycle schedule, *etc.*), being ready to start network operation.

**Figure 6.**
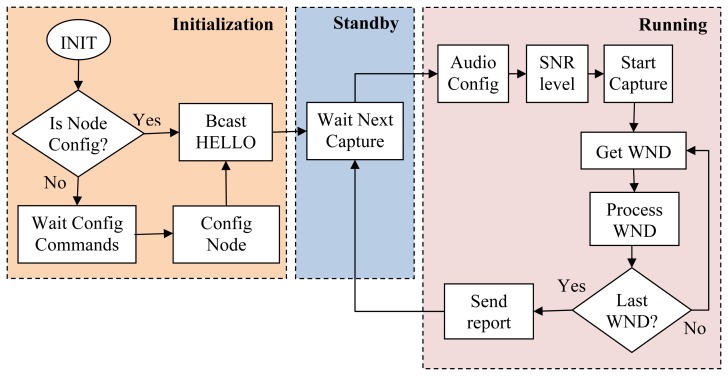
Flow diagram of the bioacoustic sensor operating software, with its three differentiated working modes: Initialization, standby and running.

Figure 6 shows, by means of a flow diagram, the different stages of the operating software installed in each sensor node. After the initialization stage, the sensor node enters a loop of running and standby modes defined by the audio capture period established at initialization stage. Thus, at the beginning of the capture cycle, the sensor node goes in running mode, where audio is captured and analyzed in real-time. When the RPW audio analysis period finishes, the sensor node delivers the corresponding report to control station, then it goes in standby mode, waiting for the beginning of the next capture cycle.

In running mode, the first operation performed by the sensor node is to configure and initialize the audio system with the parameters established at the initialization stage (sample bits, sampling rate, window size, *etc.*). Then, a Signal-to-Noise Ratio (SNR) measurement test is executed to determine the actual noise level before the audio analysis procedure; this step is fundamental for the performance of our RPW audio analysis algorithm. When the actual SNR level is known, the RPW audio analysis algorithm starts capturing audio samples in windows of fixed length (“Get WND” step at Figure 6) that may be overlapped in a predefined portion that ranges from 0% (no window overlapping) to 50% (next window will start just at the half of the current one).

Window overlapping must be carefully defined, not being too high—in order to guarantee real-time processing and low power consumption—neither too low—to avoid losing potential RPW audio segments located just between two consecutive windows. In our studies we have chosen 4096-sample fixed size windows with the minimal overlapping that prevents potential losses of RPW signals located at window edges.

Once we have captured a window of audio samples, we proceed to apply our RPW analysis algorithm. If this window contains one or more RPW audio signals, the analysis results are time stamped and stored as part of the running mode analysis report. When the last captured sample window is processed, the sensor builds a report which is wirelessly delivered to the control station, and waits for a message back indicating an acknowledgement of the successful report reception. This message may contain operational instructions, as new monitoring schedule, hardware status report required in the next duty cycle, audio configuration changes, *etc.*, for the sensor to perform just before entering the standby mode.

### 4.1. RPW Sound Model

To determine the sound model, we have used a set of segments captured with the von Laar equipment [7] by Susi Gomez and Michel Ferry (Estación Phoenix at Elche, Spain). These real-field recordings have been taken from the offshoots of severely infested adult palm trees. Most of the recordings come from *Phoenix canariensis* and *Phoenix dactylifera* species. In some audio segments, we have got general information about the infestation level, since the affected palm tree was destroyed just after getting the recordings, confirming later the presence of several RPW generations inside the palm trunks. We have classified three different kinds of sounds coming from RPW larvae: “eating”, “squealing” and “moving”. The first one corresponds to the characteristic crunch sound produced when the RPW larvae chew internal palm fibers. The second one, “squealing” is also a characteristic RPW sound but its cause is not clear. And finally, the last one corresponds to the larvae movement through galleries inside the palm trunk. For our study, we have chosen the first one, “eating”, because (1) it is the loudest sound, (2) its frequency is clearly superior to the other identified sounds in all available recordings, and (3) it is representative of the RPW larvae feeding actions in the first stages of their evolution. Consequently, we think that this one is the proper target sound to analyze in our early detection bioacoustic sensor.

**Figure 7.**
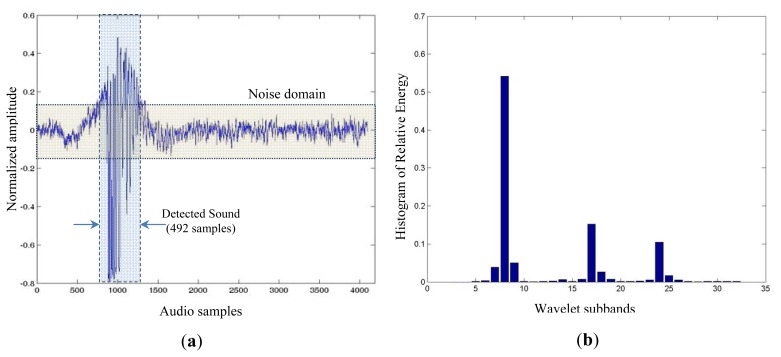
A 4096 sample window containing the RPW “eating” sound captured at 44.1 kHz sampling rate; (**a**) Temporal, and (**b**) frequency domain.

Figure 7 shows a good example of “eating” sound that was extracted from the recordings above mentioned. We can see the “eating” sound in both temporal, Figure 7(a), and frequency domains, Figure 7(b). The later was obtained from a wavelet packet transform with five decomposition levels that equally divides the input audio signal bandwidth in 32 subbands (horizontal axis). For each wavelet subband, we have computed the normalized energy of its coefficients in order to visually determine the energy distribution. As it can be seen, in Figure 7(b), there are three subbands that accumulate most of the signal energy. Thus, energy distribution across wavelet subbands may be considered a spectral fingerprint of RPW feeding activity, being an important feature to be included in our RPW “eating” sound model.

We also analyze the “eating” sample in the temporal domain, determining its main duration in terms of audio samples. We need to determine the beginning and ending positions of “eating” sound inside captured audio window using three parameters: audio signal level, signal variance, and SNR level, as shown in Figure 7(a). With the SNR level we determine the noise domain present in the captured audio, which is the actual noise level plus a 6 dB margin. The beginning of a new sound will be determined by *α* consecutive samples which value is above noise domain; meanwhile, the ending of the new sound will be determined just before *β* consecutive samples, which value falls inside noise domain. Both parameters are empirically established to properly identify potential RPW sounds. It can be seen in Figure 7(a) that the RPW “eating” temporal extension is of 492 samples (around 11 milliseconds).

Notice that not all of the “eating” samples have been recorded with the same quality as the one shown in Figure 7. There are “eating” samples with (1) different noise levels, mainly due to the recording conditions, like sound probe placement and environmental noise present at recording time, (2) different sound levels due to the distance between the source RPW and the audio probe resulting in strong, average, and weak “eating” samples, and (3) also different temporal extensions of “eating” samples. We have identified a set of more than 100 different RPW “eating” sounds in the original audio recordings employed for this purpose.

Our model is based in the following RPW “eating” sound features: (a) the energy distribution at frequency domain, and (b) the temporal features that determine the extension of the “eating” sound. First, we have manually identified all the RPW “eating” sounds from the available recordings to build the data sample space *S*. For each audio sample we have calculated its associated feature vector, *X_i_* [*sb*_1_, *sb*_2_, …, *sb*_32_, *T_ext_*], ∀ *i* = 1..|*S*|, that is composed by the wavelet subband energy distribution (*sb_i_* values) and its temporal extension (*T_ext_*). Then, we have performed a dimension reduction of the feature vectors through a vector quantization (VQ) [17] process, resulting in new set, *S̃*, of two dimensional (frequency and time) vectors, *X̃_ι_*(*f*,*t*) = *VQ*(*X_i_*[…]), ∀*i* = 1..|*S*|, where the frequency value, *f*, is obtained through a weighted average of the energy found at every wavelet subband (the weights assigned to every wavelet subband are established as a function of its relevance in the RPW “eating” spectral decomposition, as it can be observed in Figure 7(b), and the temporal dimension value, *t*, represents the temporal extension value found at the original feature vector.

We have established as the reference feature vector the one associated with the best RPW “eating” sample (high SNR level, strong and clean signal), corresponding to the one shown in Figure 7. To determine the distance of all feature vectors with respect to the reference one, we have used the Euclidean distance. The largest distance found among the quantized feature vectors and the MIN-MAX thresholds found at each dimension (frequency and time) are considered to determine whether a sound under analysis corresponds to a RPW “eating” sound.

When a new sound is detected during the audio processing of a particular sample window, we (1) obtain its feature vector, (2) perform the vector quantization, and (3) check if the resulting frequency and temporal values of the quantized vector fall between the MIN-MAX thresholds. In that case, the new sound is labeled as an RPW “eating” sound, and a score between 0 and 1 is assigned as a function of its distance to the reference feature vector. This value corresponds to the probability of being an RPW positive.

### 4.2. RPW Audio Analysis Algorithm

The RPW audio analysis algorithm is applied to every single captured window during the running mode operation of our sensor node, shown in Figure 6 as “Process WND” step. As explained above, the default size of each captured window is set to 4,096 samples. The first operation over the captured audio samples applies a Hanning filter [18] with a window size of 16 that removes high frequency noise, smoothing the captured raw audio samples. Then, we start a process to identify potential sounds that may be candidates to be analyzed as RPW “eating” sounds. Beginning at the start of a captured window we proceed through the samples to find audio segments that contain a clear sound (above 6 dB over the established SNR level) with an extension that falls between the minimum and maximum extension thresholds defined at the RPW sound model. If no potential sound is found when the end of the current window is reached, the analysis finishes without results. However, if one potential sound candidate is found, the algorithm proceeds to analyze the identified audio segment as explained above, providing the corresponding analysis results and resuming the identification of more potential candidates along the rest of the current window. This is an interesting approach, since it avoids performing full acoustic analysis to the entire window when there is no potential RPW “eating” sound candidates, saving energy and running fast enough to fit real-time restrictions.

When a potential sound is identified in the current window, the analysis algorithm performs the following tasks: (1) apply a wavelet packet transform over the audio segment with a 5-level decomposition, providing 32 frequency subbands, (2) discard the four lowest frequency subbands (from 0 to 680 Hz) when no information about “eating” sound is found in our corpora of RPW samples, (3) compute the energy distribution across all frequency subbands, and (4) obtain the corresponding feature vector to determine if the resulting frequency and temporal values of the quantized vector fall between the MIN-MAX thresholds. In that case, the sound under analysis may be classified as an RPW “eating” sound and a final analysis score value between 0 and 1 is provided, being 0 interpreted as a highly doubtful positive and 1 a completely safe positive.

Each positive found in the current window produces a report containing (1) the relative start and end sample positions, (2) the window ID number, and (3) the corresponding score value. Each report is conveniently stored in memory, so when running mode operation finishes, the list of stored window reports can be processed and wirelessly delivered to the control station. If no report is available, *i.e.*, no potential sounds detected during the running mode operation, a wireless packet is also delivered to control station as a heartbeat, indicating that the sensor node is operative and running.

## 5. Bioacoustic Sensor: Performance Evaluation

In this section, we perform a detailed evaluation of the proposed bioacoustic sensor, emphasizing the performance of the RPW audio analysis algorithm. First of all, we describe the corpora of RPW sound recordings used in the performance tests. Then, we report the experiments carried out and the methodology followed to determine the performance of our algorithm. Next, a summary of performance results is analyzed to determine the effectiveness of our proposal. In addition, we have carried out some resilience tests to settle the sensibility of our algorithm against external noise, audio sampling rate, and the number of bits per sample employed in the A/D conversion. An additional blind test considering RPW sounds, noise recordings and sounds produced by other insects belonging to the palm tree ecosystem has been developed in order to assess the performance of our detection algorithm. Finally, we have made a comparative analysis between our bioacoustic sensor and other acoustic detection systems in terms of a set of desirable features of an early RPW acoustic detection system.

### 5.1. Corpora of Sound Recordings

Michael Ferry and Susi Gómez, from the Estación Phoenix laboratory in Elche (Spain) have personally recorded several hours of RPW sounds using the von Laar RPW system [7] in different areas of the Spanish Mediterranean Rim (Granada, Malaga, Almería and Alicante). They have provided all the recordings for our study and have provided the recording conditions (audio probe placement, environmental noise, RPW infestation levels, *etc.*) and also the interpretation of the registered RPW sounds. Another set of RPW recordings has been provided by von Laar's Audio CD Small Collection Edition [19], where 13 RPW sound tracks are available, including a description of each audio track. The contents of “von Laar” audio CD has been captured with the same equipment than the Estación Phoenix audio set, but we have no information about the recording conditions. Finally, from several sound sources, we have built the third audio recording set with a selection of “non RPW” sounds that are usually present in palm trees environment, like the sound produced by palms when the wind blows, environmental noise produced by human activity (traffic, construction activity, *etc.*), and the sounds coming from other insects or birds [19]. This set is useful to test the selectiveness and resilience level of our algorithm.

### 5.2. Experimental Tests

To carry out the experimental tests, we must first know the location of every RPW single “eating” sound in the available recordings, to fix their corresponding time intervals in the audio files. Thus, we have annotated by listening tests of a trained operator (subjective tests) the locations and number of RPW “eating” sounds found in the selected audio recordings, giving also subjective information about each detected RPW “eating” sound, like strong, weak, short, several sounds at a time, *etc.* Also, we have identified non RPW sounds. This is one of the first tasks we have to do, since it is also needed for the definition of the RPW “eating” sound model explained in Section 4.1.

Once identified the location of the RPW “eating” sounds in the available recordings, we need to define the performance metrics that we are going to use to evaluate our detection algorithm. For that purpose, the performance of our detection algorithm applied to a particular audio recording is defined through the following performance metrics:

- %Positives (Pos): It determines the ratio between number of detected RPW “eating” sounds and the total number of RPW sounds found in subjective tests.
- %False Positives (FPos): It determines the ratio of detected RPW “eating” sounds that do not correspond to the real RPW sounds found in subjective tests.
- %Undetected (Undetect): It determines the ratio between the number of undetected RPW “eating” sounds and total number of RPW sounds found in subjective tests.

Additionally, for each of the above performance metrics, we have defined the following derivations that improve the analysis of performance results:

- %No_model: It determines the ratio of Pos/Fpos/Undetect RPW “eating” sounds, if we do not consider those RPW sounds that do not follow the “eating” model (*i.e.*, RPW moving sounds).
- %No_weak&short: It determines the ratio of Pos/Fpos/Undetect RPW “eating” sounds, if we do not consider those RPW “eating” sounds that are too weak or short to be detected by our model.
- %Best_case: It determines the ratio of Pos/Fpos/Undetect RPW “eating” sounds, if we do not consider those RPW sounds identified as “No_model” and “No_weak&short”.

### 5.3. Performance Results

The first set of experiments has been done over a selection of 10 audio recordings taken from the Estación Phoenix recording set, since we have all the information required to assess that recorded sounds come from RPW individuals, and we have the support from Estación Phoenix staff to identify and classify the recorded sounds. All selected audio files were recorded with a 44.1 kHz sample rate and 16 bit samples.

When processing each audio file, the first operation before analyzing audio contents is to determine the SNR level found at the whole audio recording. Once the SNR level is known, then we begin the process of the audio contents by reading the first audio window and applying our detection algorithm. The analysis results are stored in a log file for their later processing, before proceeding with the next window until the end of the audio file.

**Figure 8.**
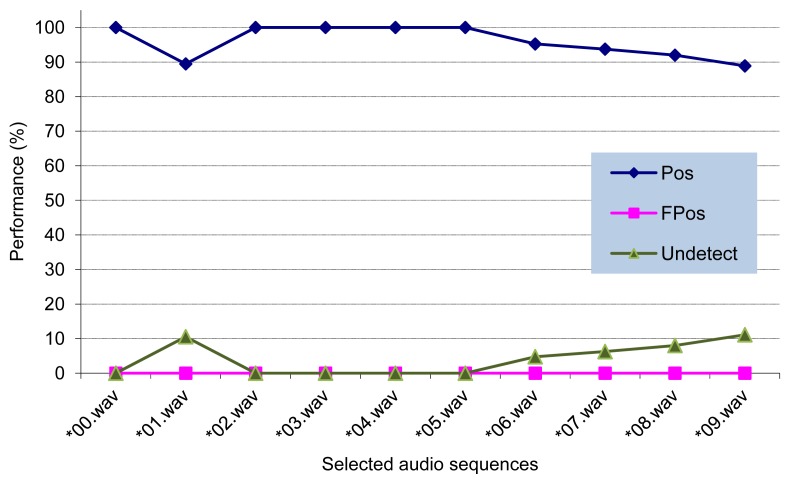
Detection algorithm performance in terms of %Positive (Pos), %False Positive (FPos), and %Undetected (Undetect) performance metrics (Best case index).

After processing each audio file, we compare the analysis results provided by our algorithm with the ones found at subjective tests, in order to obtain the values of the above defined performance metrics. Figure 8 shows the performance of our detection algorithm after processing the selected audio sequences. As it can be seen, there are no false positives, being the average detection rate around 96%.

### 5.4. Resilience of the RPW Detection Algorithm

The results presented above have been obtained with a high quality audio source that supplies 16 bit samples with a 44.1 kHz sampling rate. Besides, the SNR level found at those sequences is surprisingly high, since they were recorded from the interior of infected trunks in such a way that the environmental noise was seriously reduced. However, in real field environments, this quality may be difficult to find, so we proceed to perform several experiments with lower quality audio signals to test the resilience of the RPW detection algorithm, trying to find its performance limits.

We have employed the same set of 10 audio sequences used in previous experiments to build quality degraded versions in terms of sampling rate, bits per sample and SNR level. Consequently, we are able to evaluate our detection algorithm when working with lower quality audio signals.

**Figure 9.**
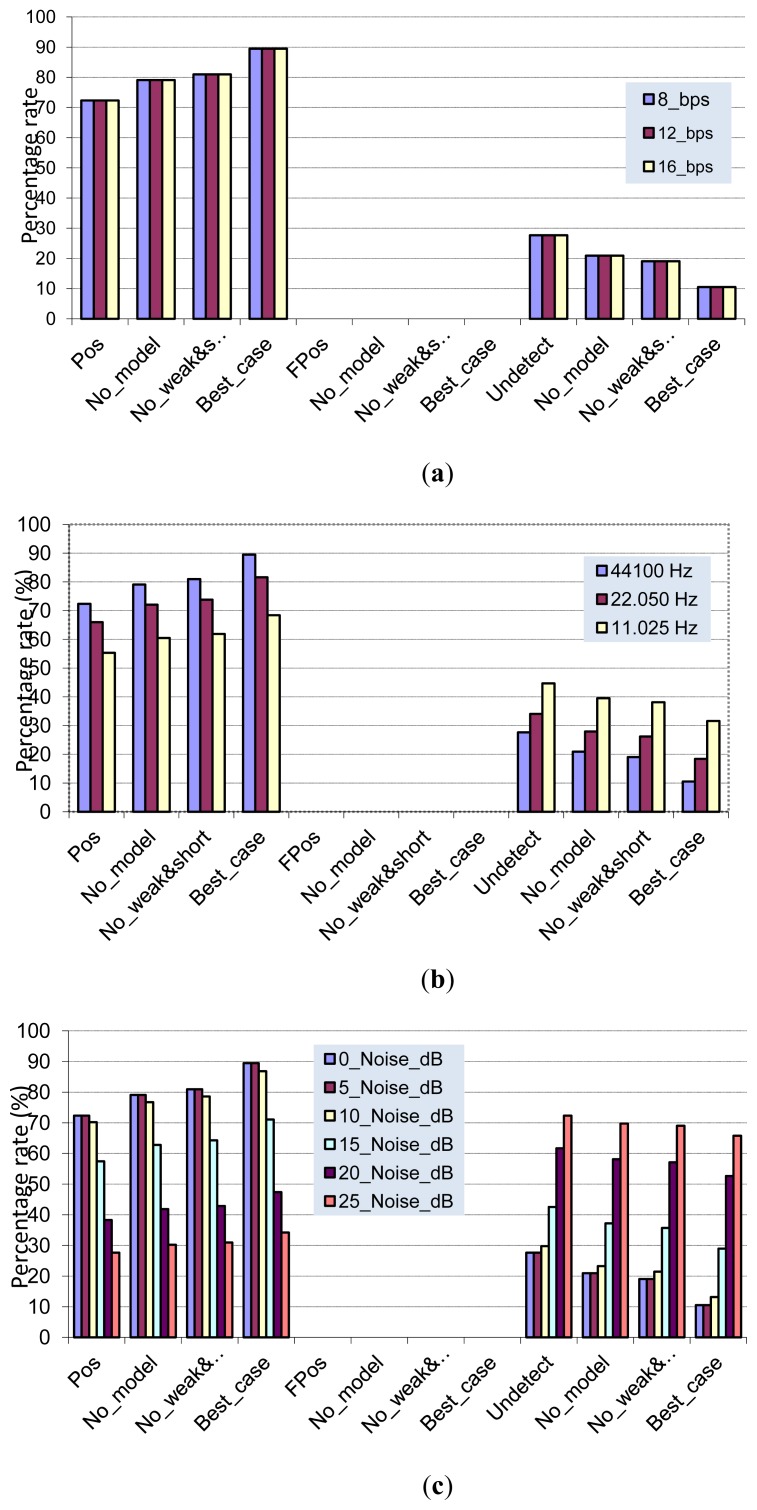
Detection performance under lower quality versions of the “*01.wav” audio sequence.

In Figure 9(a) we have built two versions of the second audio recording (“*01.wav”) by changing the number of bits per sample in a way that we have the same audio sequence with 8, 12 and 16 bit per sample. We can see that reducing the number of bits per sample (*i.e.*, increasing quantization error during audio digitalization process) has no effect in the RPW detection algorithm, since we obtain the same results. This is good news, since our algorithm is independent of the number of bits required to encode one audio sample. Then, we will use 8 bits per sample, reducing both the audio storage requirements and the computational power of the analysis algorithm.

To test these values, we have built lower quality versions by subsampling the original audio sequence by factors of 2 and 4, taking as a result the same audio sequence with 22.025 and 11.0125 kHz sampling rates. Figure 9(b) shows that reducing the sampling rate causes the reduction of our algorithm's positive detection rate, but note that no false positives are introduced. This effect may be due to the loss of both temporal and frequency information as a consequence of the subsampling process, reducing the detection rate of our algorithm up to 25%.

We employ the maximum sampling rate available depending on the resources of our sensor hardware. In particular, there is enough memory to work with capture window buffers at maximum sampling rate (capture window of 4 Kbytes of memory with a 44.1 kHz sampling rate) and the power of sensors core processor is enough to perform the proposed analysis algorithm (see Section 4.2) in real-time.

Finally, we propose another degradation of the original audio sequence by introducing a certain level of noise to observe the behavior of our detection algorithm, since the SNR level of captured audio is determinant to the success of our RPW detection algorithm. In order to generate degraded versions of the original audio sequence, we have introduced different amounts of white Gaussian noise into the original audio (5, 10, 15, 20 y 25 dBW). The results are shown in Figure 9(c), where we can observe that the proposed RPW algorithm exhibits a certain tolerance to background noise up to 10 dBW of additional noise level. However, if noise increases above 10 dBW, the performance is severely affected because the existing RPW “eating” sounds are hidden in the background noise, being difficult to detect. Nevertheless, what is most important is that a high level of background noise does not produce false positives. This behavior is mainly due to the estimation of current SNR audio level that our RPW detection algorithm performs at the beginning of each capture cycle (see Figure 6).

### 5.5. Additional Performance Tests: RPW Blind Test

Finally, in order to determine the selectiveness of our detection algorithm, we have performed a blind test driven by third party evaluators who defined a specific subset of audio sequences from the Estación Phoenix, von Laar and Non-RPW available audio sets (see Section 5.1). The resulting audio set is composed by 23 audio sequences, where 11 sequences contain at least one RPW “eating” sound, 9 sequences contain the sounds produced by other insects belonging to the palm tree ecosystem and the last three sequences contain different environmental noises (sounds produced by palms when the wind blows, sounds produced by birds or sounds coming from human activities). All of these sequences were certified by the ones who have recorded them.

The RPW blind test was arranged by third party evaluators in one session. The session consisted of reproducing the 23 audio sequences in random order with an inter-sequence interval of 5 seconds. Each audio sequence was reproduced through an audio system available in the evaluation room, our sensor placed 1 meter away from speakers. The results provided by our algorithm were displayed in real-time through a video projector; hence, the temporal correlation between the sounds heard in the room and the results shown by our algorithm could be observed on the fly. For each audio sequence, our RPW detection algorithm determines if it contains RPW activity or not.

After obtaining the analysis results of all audio sequences in the blind test, we needed to find a method to objectively measure the performance of our detection algorithm. The evaluators decided to use a binary classification scheme [20] to perform this evaluation. Binary classification is able to determine whether an individual of a population has one particular characteristic/property. It is widely used in medical testing to determine whether a patient has a certain disease; it is also used in industrial quality control procedures to evaluate if a particular product is good enough to be sold or it should be discarded because it does not reach the minimum demanded quality.

Then, our RPW detection algorithm could be considered as a binary classifier that determines whether a particular audio sequence has RPW “eating” activity. To find out the performance of our binary classifier, we need to define several statistical indexes that we arrange in the 2 × 2 matrix shown at Table 1, as the possible classification results of a particular individual.

where:

- True Positive (TP): It means that the classifier decided that the analyzed audio sequence has RPW “eating” sound activity, and it is true.
- False Positive (FP): It means that the classifier decided that the analyzed audio sequence has RPW “eating” sound activity, but it is false.
- True Negative (TN): It means that the classifier decided that the analyzed audio sequence has not RPW “eating” sound activity, and it is true.
- False Negative (FN): It means that the classifier decided that the analyzed audio sequence has not RPW “eating” sound activity, but it is false.

In Table 1, the blind test results are also shown between parentheses. The “Real values” parameter identifies the number of individuals to be classified (23 random selected audio sequences). The result of the binary classifier shows that (a) 8 out of 11 audio sequences were correctly identified as audio sequences containing RPW activity, (b) all the audio sequences with no RPW activity, *i.e.*, 12, were correctly classified, (c) 3 audio sequences with RPW activity were not detected, and (d) no sequence was erroneously classified as an RPW activity audio sequence.

There are several performance metrics that may be used to determine the performance of our binary classifier:

- Positive Predictive Value (PPV). It is defined as the relationship between the number of TPs found and the sum of TPs and FPs. This index indicates the proportion of audio sequences correctly classified as containing RPW “eating” activity.
- Negative Predictive Value (NPV). It is defined as the relationship between the number of TNs found and the sum of TNs and FNs. This index indicates the proportion of audio sequences correctly classified as not containing RPW “eating” activity.
- Sensitivity (SEN). This index determines the probability of correct classification of the individuals that contain RPW activity. It is defined as the relationship between TPs and the sum of TPs and FNs.
- Specificity (SPE). It determines the probability of correct classification of the individuals that do not contain RPW activity. It is defined as the relationship between TNs and the sum of TNs and FPs.
- Matthews Correlation Coefficient (MCC). It evaluates the quality of the binary classifier by means of expression (1). This index provides a value between –1 and 1, where 1 should be interpreted as a perfect classifier and –1 shows that the classifier performs predictions just opposite to the desired ones. An MCC value of cero will determine a completely random classifier:
  (1) $$\text{MCC}=\frac{\text{TP}.\text{TN}-\text{FP}.\text{FN}}{\sqrt{\left(\text{TP}+\text{FP}).(\text{TP}+\text{FN}).(\text{TN}+\text{FP}).(\text{TN}+\text{FN}\right)}}$$

Applying these performance indexes with the results obtained in the blind test, the value of the above performance metrics are the following: PPV = 100%; NPV = 80%; Sensitivity = 72%; Specificity = 100%; MCC = 0.76.

The way we interpret the performance of our RPW detection algorithm (binary classifier) depends on the detection specifications demanded by the application. Thus, it is of critical importance to avoid false positives, since it would cause the activation of the corresponding protocol over the area where the alerting palm tree is located, with the corresponding costs and the risk of removing healthy palm trees. Therefore, the RPW detection algorithm should provide as high PPV and Specificity metric values as possible. In our case, after performing the blind test we obtained the maximum performance score on both metrics.

Although no so critical as avoiding false positives, the RPW detection algorithm should also have a good detection rate, trying to reduce as much as possible the total number of false negatives. This would be achieved with high values of NPV and Sensitivity metrics, as those achieved by our RPW detection algorithm.

### 5.6. Comparative Analysis with other RPW Acoustic Detection Systems

Finally, we have performed a comparative analysis of our acoustic sensor proposal with other proposals from literature. We have taken into account several features that we consider of interest: Average Detection Rate (ADR), False Positives Rate (FPR), Continuous Monitoring capability (CM), Unattended Operation (UO), Monitoring Costs (MC), and Computational Complexity (CC). In Table 2, we summarize the information from different RPW acoustic detection systems that we have analyzed from the literature, observing that our proposal is not so efficient in terms of average RPW detection rates than other proposals, but it has other interesting characteristics (*i.e.*, continuous monitoring) that make it an interesting choice for remote sensing palm tree orchards.

Although, we have no information about the economic cost of other acoustic RPW detection systems (some of them are only software proposals), we estimate the overall cost of our acoustic RPW sensor. Our estimation is based on the economic cost of (a) materials, (b) Printed Circuit Board (PCB) production, (c) electronic components, and (d) the assembly of all the sensor parts.

Taking into account all of these items, a prototype of our acoustic RPW sensor would be under 200 Euros. This is the economic cost of each of the four prototypes we have built; however, for larger production orders the cost per unit should be significantly reduced. When comparing the estimated costs of our RPW acoustic sensor with those from von Laar or AED2000L acoustic systems, our proposal becomes very competitive and attractive for practical RPW monitoring deployments.

## 6. Conclusions

During the last two decades, the Red Palm Weevil (RPW), also known as *Rynchophorus Ferrugineus* Oliv., has become one of the most dangerous threats to the palm trees in most parts of the world. Its early detection is difficult to assess since the infected palm trees do not show visual evidences until it is too late for the plant to recover. For this reason, the development of efficient early detection mechanisms is critical for the design of efficient RPW pest management systems.

In this context, we have presented a bioacoustic sensor able to efficiently detect the sounds produced by RPW larvae after the first infestation stages. Up to our knowledge, all of the acoustic detection systems proposed in the literature require an *in-situ* audio analysis, so: (1) continuous unattended monitoring is not supported, and (2) the monitoring costs are high, being directly proportional to the required monitoring frequency. To verify the performance of our detection algorithm we have driven a large set of experimental tests using several hours of certified audio recordings. The results show that our proposal achieves acceptable detection rates, it has certain resilience with respect to environmental noise, and it is highly selective, being able to efficiently discriminate the sounds produced by other insects.

Therefore, our bioacoustic sensor proposal copes with the restrictions above mentioned in order to provide a low-cost monitoring system with a fast detection response that exhibits the following capabilities: (1) high RPW detection rates (over 90%) with no false positives under the experimental tests we have carried out; (2) periodic audio monitoring following an user-defined schedule; (3) it works autonomously with a solar-based power unit for a long period of time (potentially eternal); (4) no maintenance is required after installation; (5) the installed bioacoustic sensors may form a wireless sensor network with a coverage from little orchards to large plantation extensions.

Our future work will be focused on the deployment of our RPW bioacoustic sensors to form a wireless sensor network in palm tree orchards. This implies the development of network software that will allow a reliable communication of each sensor with the control station. Also, we will develop the control station software that will receive the analysis reports from the installed sensors in order to process and conveniently store them with the corresponding side information (palm ID, geolocation info, timestamp, report summary, *etc.*). All the information at the control station may be accessed through Internet, allowing supervisor to remotely check the palm trees status in real-time through a nice and intuitive graphical user interface. Then, a complete monitoring tool would be available to protect palm trees from RPW pest, providing fast detection response, continuous monitoring activity and reducing the monitoring costs when compared to in-situ human-operated monitoring proposals.

## Figures and Tables

**Table 1.** Binary classifier defined by a 2 × 2 matrix with the obtained results in the test.

	**Real values (23)**	
**Classifier results**	True Positive (8)	False Positive (0)
	True Negative (12)	False Negative (3)

**Table 2.** Comparison among different RPW acoustic detection systems.

	**ADR**	**FPR**	**CM**	**UO**	**MC**	**CC**
Our proposal	90%	0%	Yes	Yes	Low	Low
[10]	97%	5.5%	No	No	High	Low
[14]	99.1%	n/a	No	Yes	Avg	High
[15]	98%	1.5%	No	Yes	Avg	Avg
